# “Letting Go and Staying Connected”: Substance Use Outcomes from a Developmentally Targeted Intervention for Parents of College Students

**DOI:** 10.1007/s11121-023-01520-6

**Published:** 2023-03-18

**Authors:** L. G. Hill, M. Bumpus, K. P. Haggerty, R. F. Catalano, B. R. Cooper, M. L. Skinner

**Affiliations:** 1 Department of Human Development, Washington State University, Pullman, Washington USA; 2 Social Development Research Group, University of Washington, Seattle, Washington USA

**Keywords:** Emerging adulthood, Substance use, Parent-student intervention, Prevention

## Abstract

We present results of a randomized, controlled, efficacy trial of a handbook intervention for parents of first-year college students. The aim of the interactive intervention was to decrease risk behaviors by increasing family protective factors. The handbook, based in self-determination theory and the social development model, provided evidence-based and developmentally targeted suggestions for parents to engage with their students in activities designed to support successful adjustment to college. We recruited 919 parent-student dyads from incoming students enrolled at a university in the U.S. Pacific Northwest and randomly assigned them to control and intervention conditions. We sent handbooks to intervention parents in June before students’ August matriculation. Research assistants trained in motivational interviewing contacted parents to encourage use of the handbook. Control parents and students received treatment as usual. Participants completed baseline surveys during their final semester in high school (time 1) and their first semester at college (time 2). Self-reported frequency of alcohol, cannabis, and simultaneous use increased across both handbook and control students. In intent-to-treat analyses, odds of increased use were consistently lower and of similar magnitude for students in the intervention condition than in the control condition, and odds of first-time use were also lower in the intervention condition. Contact from research assistants predicted parents’ engagement, and parent and student report of active engagement with handbook predicted lower substance use among intervention than control students across the transition to college. We developed a low-cost, theory-based handbook to help parents support their young adult children as they transition to independent college life. Students whose parents used the handbook were less likely to initiate or increase substance use than students in the control condition during their first semester in college.

ClinicalTrials.gov Identifier: NCT03227809

**Supplementary Information:**

The online version contains supplementary material available at 10.1007/s11121-023-01520-6.

## Introduction

It is well established that first-year college students living away from home frequently either initiate or increase their use of alcohol and drugs (Hingson et al., 2017; Scott-Sheldon et al., 2014; Substance Abuse & Mental Health Services Administration, 2020), especially during the first few weeks of college (Auerbach & Collins, 2006; Riordan et al., 2015). The transition to the college environment is a developmental milestone usually marked by increased exposure to opportunities for alcohol and substance use, decreased structure, identity exploration (Arnett, 2000), loss of previous support structures, and reduced supervision of students’ behavior (Schulenberg & Maggs, 2002). Relatedly, first-year students are often at high risk for experiencing harms associated with alcohol and substance use, including high-risk sex behaviors (Bailey et al., 2008, 2011; Vail-Smith et al., 2010), academic difficulties (Arria et al., 2018), disciplinary or legal sanctions, accidents, death, and mental and physical health problems (Hingson, 2010). In response to these realities, university personnel have employed numerous intervention efforts targeting students directly to reduce the likelihood of harm resulting from student alcohol and substance use and misuse (Plotnikoff et al., 2019).

Parents and caregivers are another potentially promising agent in university prevention efforts (Mallett et al., 2019; Turrisi et al., 2001). Parents generally remain an important influence on their children’s decision making, even after the transition to college (Arnett, 2000; Messler et al., 2016; Settersten, 2012), and parent-student communication has been established as a protective factor with regard to student alcohol use (Madkour et al., 2017; Ryan et al., 2010; Sher & Rutledge, 2007; Small et al., 2011). In addition, cell phones and social media create virtually limitless opportunities for parent-student communication, and many university students report that they value conversations with parents (Pizzolato & Hickien, 2011).

On the other hand, the role of parents in the lives of their college students is often ambiguous, because some roles traditionally considered parental, such as responsibility for decision making, health, daily schedules and logistics, problem solving, and finances, are increasingly transferred to their young adult children (Lowe & Dotterer, 2018). Parents usually have fewer opportunities for face-to-face interaction than before the college transition and as a result may feel less equipped to provide guidance about issues that arise in their student’s life. In addition, parents may perceive that transition to adulthood means that parents have less authority for monitoring or socialization of their student’s behavior (Darlow et al., 2017). Finally, parents may not have models for effective parenting during this developmental period, especially given the plethora of negative media stereotypes about “helicopter parents” and messages to back off (Kloep & Hendry, 2010), without guidance on how to both let go and stay connected.

The proximal target of the handbook intervention was parents’ interactions with their students. We aimed to provide parents with strategies to meet the needs of this developmental period. The handbook includes suggested conversations and activities for parents to engage in with their students. These were designed to increase parents’ understanding of the need to (1) support autonomy growth by coaching and asking questions instead of advising; (2) support their students as they navigate myriad new situations; and (3) have reciprocal conversations about values and expectations and continue checking in about substance use, health behaviors, peer relationships, and academics.

These approaches to parenting in the transition to college are protective. They help parents navigate their continued roles in encouraging and monitoring students at a distance while still supporting their young adult students’ increasing autonomy. The handbook, by providing examples and opportunities to practice meaningful conversations, (1) helps parents gain respect for and support of students’ autonomy growth by encouraging a focus on the students’ values-based decisions rather than on telling students what to do; (2) emphasizes the continued need for emotional support of young adult children; and (3) provides a developmentally appropriate approach to parental monitoring.

We hypothesized that by using the handbook, parents would meet the changing developmental needs of their students as they moved away from home, and that this in turn would decrease likelihood of students’ use of alcohol and cannabis in the high-risk period immediately following transition to college. Evidence-based interventions for parents during this developmental period are scarce relative to programming that focuses either on parents of adolescents or on college students themselves; in a special section of *Prevention Science,* Stormshak and her colleagues noted that “it is remarkable that family-centered prevention and intervention seldom extends into emerging adulthood” (2019, p. 322). The interventions that do exist for parents of young adults are mostly didactic, and they focus on providing parents with information about college student alcohol use and strategies for talking to their student about alcohol as a means to reducing student alcohol misuse and harm (Catalano et al., 1996; Turrisi et al., 2001). However, they do not address underlying factors such as the nature of the parent-student relationship and its importance in the developmental context of the student leaving for college.

The small existing body of research on interventions with parents of college students generally falls into one of two categories. The first, exemplified by the pioneering work of Turrisi and his colleagues, involves a didactic approach in which parents are provided with materials on how to talk to their student about alcohol use and misuse at college, with a particular focus on challenging myths about drinking and making parental expectations about student drinking clear (Donovan et al., 2012; Ichiyama et al., 2009; Testa & Livingston, 2009; Turrisi & Ray, 2010; Turrisi et al., 2001). Results from these studies have generally shown positive and modest effects on college student alcohol use and misuse. The second approach to parent interventions with their college student offspring involves giving normative feedback for parents, online or in person using clickers at an orientation session, about students’ alcohol use and attitudes regarding drinking (e.g., Hummer et al., 2013; Labrie et al., 2014). Results of these studies have shown that, among other findings, providing normative feedback to parents predicts lower perceived parental approval of drinking and, in turn, less student alcohol use in general as well as less student heavy episodic drinking (LaBrie et al., 2016).

Here, we present a different approach to parent-based interventions with college students. Our intervention, *First Years Away from Home: Letting Go and Staying Connected*, is theoretically grounded and integrates principles from two well-established theories that have helped to identify the mechanisms linking parenting characteristics and practices to child and youth outcomes. The first, self-determination theory (Ryan & Deci, 2000), focuses on individuals’ motivations and points to the importance of relationships that provide support for one’s autonomy during key developmental periods such as the transition to college. The second, the social development model (Catalano et al., 1996), provides parents with strategies that promote bonding and support effective and developmentally appropriate parenting strategies to monitor safety and set age-appropriate expectations. These two theories posit complementary models of behavior, and both emphasize the need for, and protective nature of, three domains of parenting: relatedness, structure and opportunities for responsibility, and increasing growth of autonomy. The forms that parental support takes changes during these years — for example, monitoring of student behavior occurs at a distance and at a time when students are both learning to make their own decisions and being exposed to many new opportunities. Thus, parents should ideally adjust their monitoring to accommodate new realities.

## Letting Go and Staying Connected

*First Years Away from Home: Letting Go and Staying Connected* is delivered via a handbook in paper form that families receive by mail early in the summer prior to the student starting at college. Parents and students are encouraged to work through the 14-page handbook content together periodically throughout the summer; because the intervention is self-directed, family members can also revisit key sections once the student has left for college. Specifically, our intervention gives parents some background information on emerging adulthood, describes important challenges and opportunities that students experience across the transition to college, and provides a brief introduction to theoretical and empirical rationales for why parents remain important in the lives of their college student offspring. We introduce the idea that during the college years, parents have three important roles: *cheerleader* (providing emotional support); *coach* (providing autonomy support, helping students clarify their values); and *safety monitor* (communicating clear behavioral expectations, especially around issues of potential harm, and checking in with their students about health and risk behaviors). The cheerleader role corresponds to the construct of relatedness in self-determination theory and of prosocial bonding with parents in social development theory. The coach role supports students in development of autonomy skills (self-determination theory), and the safety monitor role corresponds to family management skills (social development) and communicating clear expectations leading to increased competence (self-determination).

Handbook content is interactive and features some activities that parents can do alone and others that parents and students can undertake together to (a) help parents practice the basic principles of each parenting role, and (b) facilitate effective parent-student communication and perspective-taking related to multiple aspects of students’ lives, including social, academic, and health behaviors. The handbook provides several scenarios (e.g., poor grades first semester; roommate troubles; parties for a home football game) and asks parents to consider their primary role or roles in each scenario and think through the implications for how they communicate with their student in that situation. For example, in the “poor grades” scenario, parents take on the roles of both cheerleader, emphasizing that they believe in the student’s ability to achieve their goals, and of coach, helping the student problem-solve and asking questions to help the student clarify possible approaches to improve grades. In the “home football game” scenario, parents take on the role primarily of safety monitor, helping the student think through potential risks of partying and stating their own expectations about the student’s substance use.

As a foundation for this type of conversation, one of the intervention exercises that family members undertake together is an expectations card sort. Parents and students each have their own identical deck of cards, and each card lists one issue (such as “communication with parents” or “using alcohol”, or “grades and academic performance”). Parents and students then separately sort each card to indicate whether their expectations regarding that topic are clear, unclear, or if the topic is a non-issue. They are then able to compare each other’s ratings and identify discrepancies and issues for which more discussion may be warranted before the student leaves for college. Similarly, parents and students each have another set of cards with values listed on them (e.g., “healthy eating, exercise”; “choosing a career or major”, or “checking in with parents”). Parents and students separately sort cards into four piles according to personal level of importance; they then compare and discuss differences and similarity in values. A practical activity is the Financial Readiness Checklist, in which parents and students decide who is financially responsible for items such as laundry, travel back and forth to college, books, and so on. Parents are encouraged to engage in those conversations using strategies that are introduced in the discussion of the three parenting roles. The activities give family members clarity about one another’s values, a focus for discussing issues together, and also provide context for reinforcing developmentally informed parenting roles.

An enhanced condition includes booster texts beginning in students’ first semester. Text messages refer to parent roles described in the handbook and suggest communicating with their child about specific topics. Messages may be timed to coincide with especially stressful events (such as midterms), and others may be timed to remind parents about significant university events (such as homecoming, “Dad’s Weekend,” and Halloween) associated with high numbers of sanctions for substance-use violations. We did not expect to see any effects of the text boosters in the first semester, because most text messages were not sent until after the first-semester data collection occurred. Thus, for purposes of this paper we collapsed Handbook and enhanced Handbook conditions.

In numerous ways, then, our intervention differs from other parent-student interventions for this age group: it is derived from developmental theory, focused more broadly on parenting strategies than on reducing substance use risk, and its content provides rich opportunities for conversations about values and expectations to emerge naturally while parents and their young-adult children are engaged in a task together. Although there was little content about substance use, we hypothesized that the handbook would have protective effects on student substance use during fall of the first semester. Our approach represents a promising step in this field, given that interactive programming has been demonstrated to be more effective than didactic approaches in reducing adolescent alcohol and substance use (Lize et al., 2017). The current study is part of a larger randomized, controlled trial funded by the National Institute on Drug Abuse (NIDA) comparing efficacy of the parent handbook described above with a treatment-as-usual control group.

## Method

### Recruitment and Sample

We recruited participants during early spring of two consecutive years, in the final semester of students’ senior year in high school. Each cohort participated for two full years, from the spring of recruitment through the spring of their second year (fourth semester) in college. We controlled for potential cohort effects by using cohort as a covariate in outcome analyses. The population from which we drew our sample comprised all students admitted to two consecutive first-year university classes, at a single university in the Pacific Northwest region of the United States (CONSORT diagram in Supplementary materials). To reach our target sample size of 900, we randomly selected from the admitted population the study sampling pool of 1567 students who met eligibility criteria (first time attending college, younger than age 21, living in the USA, English speaking, beginning college in fall semester, not living in the university town). We then randomly allocated those students to a control condition, an intervention condition (Handbook), and the enhanced intervention (Handbook plus booster texts).

We recruited parent-student dyads, contacting students and their parents or caregivers first by letter and email, with follow-up telephone calls explaining the study. To be enrolled in the study, both the student and one parent or caregiver had to agree to participate and then had to complete a baseline survey. Our final sample size was slightly more than our target (*N* = 919) to achieve a sample representative of the university demographics (Control *n* = 309; Handbook *n* = 610). After the first two waves of data collection, one dyad withdrew from the university and requested that their data be withdrawn. Thus, the final sample size was 918. Additional details on the study sample and protocol can be found in Cooper et al. (2020). The current paper covers results from baseline (spring semester of high school) to first semester in college.

The student sample was representative of the university’s first-year student population: 49.6% female and 32.5% first-generation students. (At the time we recruited for the study, the administrative database allowed for only binary gender identification.) Fifty-nine percent (59%) of students reported their race/ethnicity as White, 17.4% as Hispanic/Latinx, 4.5% as Black/African American, 1% as American Indian/Alaska Native, 5.1% as Asian, 0.7% as Hawaiian/Pacific Islander, and 11.3% as two or more races. Average age at recruitment was 18.2 (SD = 0.36, range = 16.7—19.6).

Eighty-six percent (*n* = 522) of parents selected only one race category and, of those, 82% identified as White only, 6% identified as Hispanic only, 5% identified as Black only, fewer than 1% identified as Native only, and 7% identified as some other race. Nine percent selected two or more race categories and 5% did not report race information. We did not collect data on parent age. Nearly 78% of parent/caregiver members of the dyads were mothers and 21.9% were fathers, with the remaining 0.5% described as a sibling (*n* = 1), aunt (*n* = 1), or stepparent (*n* = 3). Seventy-three percent of students reported living with both parents at baseline.

There were no statistical differences between groups at baseline on the substance use outcomes. Retention from baseline to first semester was 96% for parents and 94% for students. There was no differential attrition across conditions between the baseline and follow-up surveys.

### Procedure

Each parent or caregiver and student independently completed a baseline survey in spring of students’ senior year in high school. Then, early in the summer, we sent copies of the handbook to parents in the intervention conditions. As an implementation strategy, we hired graduate student research assistants to increase parent engagement by calling intervention parents to encourage them to read the handbook and complete the activities. To facilitate conversations with parents about using the handbook, research assistants received 8 h of training on a standardized call protocol from one of the investigators and one of the PIs approximately 1 month before the handbooks were mailed to families. The training provided information about the handbook, motivational interviewing skills, and opportunities to practice using motivational interviewing. After completing at least three practice calls with the research team and being approved by the senior family outreach supervisor, each research assistant was assigned an average of 48 families. Once handbooks had been sent, research assistants began calls to parents in the intervention condition and successfully contacted 76% (*n* = 464) of them. The first contact with parents was by phone, and research assistants used motivational interview principles in those calls to engage parent interest. The number of additional reminders by phone, email, or text varied according to parents’ availability, contact preferences, and willingness to talk. Participants in the control condition received no intervention beyond what was offered by the university (e.g., mandated parent orientation and student attendance at trainings on substance use and other risk behaviors).

### Measures

#### Outcome Measures

We selected standard measures of substance use that are used in statewide surveys in Grades 6—12 (Washington State Department of Social and Health Services, 2017) and in national US surveys such as the Monitoring the Future survey (Schulenberg et al., 2021). At both baseline (spring of senior year in high school) and follow-up (early fall of first semester in college), students responded to questions about past 30-day use of alcohol, cannabis, and simultaneous use of alcohol and cannabis: “During the past 30 days, on how many occasions have you used [alcohol/marijuana /alcohol and cannabis (e.g., marijuana, hashish) at the same time so that the effects overlapped (i.e., cross fading)]?” (Washington State Department of Social and Health Services, 2017). We also asked how many times students had had eight or more (for females) or 10 or more (for males) alcoholic drinks in a row over the past 30 days (heavy episodic drinking), and how many times students had had four or more (females) or five or more (males) alcoholic drinks in a row in the past 2 weeks (binge drinking) (National Institute on Alcohol Abuse and Alcoholism, n.d.).

Consistent with national and state surveys, response scales for each item were on a 7-point ordinal scale ranging from 0 occasions to 40 or more occasions. Because distributions were heavily skewed, we dichotomized each of these variables into “none in past 30 days” (or “over the past two weeks” for binge drinking) and “at least once in past 30 days/two weeks.” Beginning with cohort 2, we included the additional option of “I have never done this” and dichotomized responses to this item into a lifetime use variable (“never used” or “used”); this option was inadvertently omitted from the survey administered to cohort 1. thus, we have information on lifetime use of substances for cohort 2 but not for cohort 1.

#### Covariates

We included baseline (spring of senior year in high school) substance use, cohort, minoritized race/ethnicity (as a dichotomized variable with race/ethnicity collapsed for minoritized students due to small numbers in most categories), first-generation college status (i.e., neither parent reported attending college), and gender as covariates in all models.

#### Implementation Measures

We tested whether calls from research assistants were successful in prompting parents to engage with the handbook. This dichotomous measure was coded as 0 for no contact and 1 for contact with parents. In the fall data collection, we assessed parent engagement by asking how many hours they spent reading the handbook and how many hours they spent actually doing handbook activities with their student (both items rated on a 9-point ordinal scale ranging from “none, I did not read any of the handbook/do any of the activities” to “more than 10 h”). We also asked parents how useful and how engaged they thought their student was with the activities (both items rated on a 4-point response scale ranging from “not at all” to “extremely”). Students reported whether they remembered their parents reading the handbook and whether they did any of the activities together (responses of “yes” and “no”).

### Analytic Approach

We examined prevalence of each outcome at baseline and in fall semester. We then used intent-to-treat logistic regression models to test effects of the intervention on change in use for each outcome. We also used logistic regression models to examine associations between phone contact with parents with parent-reported parent engagement, and parent-reported implementation variables with student substance use outcomes.

## Results

### Prevalence of Substance Use

Raw frequencies showed that unadjusted prevalence of all categories of substance use increased substantially from high school to first semester at college but that prevalence in the intervention condition was substantially lower than in the control condition for all substance use outcomes after entry to college (Table 1). Similarly, logistic regressions (Tables 2 and 3), controlling for baseline use, cohort, minority race/ethnicity, first-generation college-going status, and gender, showed that early in the fall semester after arriving at college, the odds of 30-day alcohol use among intervention students were 33% lower than among control students (OR = 0.67, *p* = 0.02), 28% lower for cannabis use (OR = 0.72, *p* = 0.05), 26% lower for simultaneous use of alcohol and *cannabis (OR* = *0*.74, *p* = 0.09), 27% lower for binge drinking (OR = 0.73, *p* = 0.05), and 28% lower for heavy episodic drinking (OR = 0.72, *p* = 0.07).

**Table 1:** Prevalence of substance use outcomes

Outcome	Baseline		Follow-up		Increase from baseline to follow-up	
	Control *n* = *309*	Handbook *n* = *609*	Control *n* = *294*	Handbook *n* = *569*	Control *n* = *294*	Handbook *n* = *569*
*Past 30-day prevalence*						
Alcohol use	31.07%	35.30%	69.73%	62.92%	38.66%	27.62%
Cannabis use	14.89%	17.73%	37.41%	33.92%	22.52%	16.19%
Simultaneous use	7.77%	11.00%	25.85%	24.25%	18.08%	13.25%
Binge drinking^*a*^	8.74%	12.81%	50.00%	45.87%	41.26%	33.06%
Heavy episodic drinking	13.59%	15.11%	26.19%	23.90%	12.60%	8.79%
	Control *n* = *192*	Handbook *n* = *381*	Control *n* = *183*	Handbook *n* = *350*	Control *n* = *183*	Handbook *n* = *350*
*Lifetime prevalence*^*b*^						
Alcohol use	59.38%	54.07%	78.69%	68.00%	19.31%	13.93%
Cannabis use	28.65%	32.55%	57.92%	47.71%	29.27%	15.16%
Simultaneous use	18.23%	21.78%	44.81%	37.71%	26.58%	15.93%

Controlling for cohort, minoritized race/ethnicity, first generation, and sex

^a^Past 2 weeks

^b^This information was collected for cohort 2 only. The sample size reflects the number of students who reported no lifetime use at baseline. Cohort not included as a covariate

**Table 2:** Logistic regression: odds ratio of increase in substance use prevalence from baseline to first semester (*N* = 862)

	Alcohol use				Cannabis use				Simultaneous use			
Predictor	OR	*p*	Lower limit	Upper limit	OR	*p*	Lower limit	Upper limit	OR	*p*	Lower limit	Upper limit
Baseline use	9.41	< 0.001	6.03	14.67	13.80	< 0.001	8.59	22.17	9.87	< 0.001	5.85	16.66
Cohort	1.29	0.15	0.91	1.81	0.85	0.35	0.60	1.20	0.85	0.39	0.59	1.23
Minoritized race/ethnicity = 1	0.91	0.56	0.65	1.27	1.03	0.85	0.73	1.46	0.84	0.37	0.58	1.22
First generation = 1	0.64	0.01	0.46	0.89	0.84	0.33	0.60	1.19	0.90	0.59	0.63	1.30
Female = 1	1.39	0.04	1.01	1.91	0.76	0.10	0.55	1.05	0.67	0.03	0.48	0.95
Handbook = 1	0.67	0.02	0.48	0.94	0.72	0.05	0.52	1.00	0.74	0.09	0.52	1.05

Controlling for cohort, minoritized race/ethnicity, first generation, and sex

**Table 3:** Logistic regression: odds ratio of increase in substance use prevalence from baseline to first semester (*N* = 862)

	Binge drinking				Heavy episodic drinking			
Predictor	OR	*p*	Lower limit	Upper limit	OR	*p*	Lower limit	Upper limit
Baseline use	18.24	< 0.001	8.26	40.29	7.93	< 0.001	5.05	12.43
Cohort	1.95	0.28	0.87	1.64	1.33	0.16	0.90	1.96
Minoritized race/ethnicity = 1	0.64	< 0.01	0.47	0.88	0.58	< 0.01	0.40	0.85
First generation = 1	0.91	0.55	0.67	1.24	0.84	0.38	0.58	1.23
Female = 1	1.15	0.35	0.86	1.55	0.54	< 0.001	0.38	0.77
Handbook = 1	0.73	0.05	0.54	0.99	0.72	0.07	0.50	1.03

Controlling for cohort, minoritized race/ethnicity, first generation, and sex

Baseline use strongly predicted all types of use in the first semester of college. Minoritized students were less likely than white students to report binge drinking and heavy episodic drinking. First-generation students were less likely to report alcohol use, and females were more likely than males to report alcohol use but less likely to report heavy episodic drinking.

### Initiation of Substance Use

Initiation of substance use shows the same pattern: a sharp increase in initiation across alcohol, cannabis, and simultaneous use (Table 4) (Binge and heavy episodic drinking are not presented because of skip patterns for those who reported no lifetime use at all at time 1.) Logistic regression models, controlling for baseline use, minoritized race/ethnicity, first-generation college-going status, and gender, showed that the handbook intervention strongly predicted continued abstention from substance use from spring to fall (see Table 4). In their first semester of college, students in the intervention condition who at baseline reported never having used alcohol/cannabis had 55% lower odds than control students of initiating use of alcohol (OR = 0.45*, p* = 0.008), 49% lower odds of initiating cannabis use (OR = 0.51, *p* = 0.005), and 44% lower odds of initiating simultaneous use of alcohol and cannabis (OR = 0.56, *p* = 0.01).

**Table 4:** Logistic regression: odds ratio of initiation of first substance use between baseline and first semester

	Alcohol use *n* = 236				Cannabis use *n* = 370				Simultaneous use* n* = 427			
Predictor	OR	*p*	Lower limit	Upper limit	OR	*p*	Lower limit	Upper limit	OR	*p*	Lower limit	Upper limit
Minoritized race/ethnicity = 1	0.88	0.64	0.51	1.52	0.91	0.68	0.57	1.44	0.88	0.58	0.57	1.38
First generation = 1	0.81	0.45	0.46	1.41	0.98	0.95	0.61	1.58	1.06	0.81	0.67	1.68
Female = 1	0.94	0.83	0.54	1.64	1.04	0.86	0.65	1.66	0.95	0.283	0.60	1.50
Handbook = 1	0.45	0.008	0.25	0.81	0.51	0.005	0.32	0.82	0.56	0.01	0.35	0.88

This information was collected for cohort 2 only. The sample size reflects the number of students who reported no lifetime use at baseline

### Handbook Implementation Check

#### Use of the Handbook

Eighty percent (80%, *n* = 439) of parents assigned to the handbook condition reported reading at least some of the handbook or doing a handbook activity. Of those, 76% reported spending an hour or more reading the handbook. Most parents (53.7%) who reported engaging at all with the handbook spent 1 to 2 h reading, and 22.1% spent 3 h or more reading. Sixty-nine percent (69.6%) of them reported spending an hour or more doing the activities, with an average number of 3.19 activities completed (SD = 2.12) out of a possible five.

A substantial majority (88.7%) of parents who engaged reported that the handbook had been at least somewhat useful for their students, with 33.5% finding it very or extremely useful. Nearly half of parents (48.7%) who did activities reported that their student was “extremely” or “very” engaged in them, with another 47% reporting that students were “somewhat” engaged. Only 4.2% reported that their student was “not at all engaged.” In logistic regression analyses, research assistant contact with parents predicted use of the handbook (B = 0.72, SE = 0.19*, p* < 0.001).

Consistent with parent report, most students (74.6%) remembered their parents reading the handbook, and 56.5% said they did one or more activities from the handbook with a parent. Eighty-nine percent (89%) remembered their parent receiving the handbook, indicating a high level of awareness of the handbook and some exposure to it.

#### Association of Parent Engagement with Outcomes

Parent report of perceived usefulness for their student was associated with lower odds of alcohol (OR = 0.75, *p* = 0.09), cannabis (OR = 0.57, *p* = 0.004), simultaneous use (OR = 0.70, *p* = 0.07), and binge drinking (OR = 0.73, *p* = 0.06) but not with heavy episodic drinking (OR = 0.82, *p* = 0.33) (Tables 5 and 6). Parent perception of students’ engagement with the handbook was also consistently associated with lower odds of past 30-day substance use (alcohol: OR = 0.72, *p* = 0.04; cannabis: OR = 0.72, *p* = 0.06; simultaneous use: 0.72, *p* = 0.08). In contrast, parent report of time spent simply reading the handbook was not strongly associated with past 30-day use of alcohol (OR = 0.85, *p* = 0.22), cannabis (OR = 0.91, *p* = 0.52), simultaneous use (OR = 0.89, *p* = 0.46), or binge drinking (OR = 0.91, *p* = 0.45), but it was moderately associated with heavy episodic drinking (OR = 0.79, *p* = 0.09), nor was it associated with initiation of alcohol (OR = 1.02, *p* = 0.92) or cannabis use (OR = 0.92, *p* = 0.57).

**Table 5:** Logistic regression: odds ratios of associations between parent engagement and T2 (fall semester) alcohol/cannabis outcomes

		Alcohol Use				Cannabis Use				Simultaneous Use			
*Predictor*	n	OR	*p*	Lower limit	Upper limit	OR	*p*	Lower limit	Upper limit	OR	*p*	Lower limit	Upper limit
*Parent report* (*n* = 437)													
Useful to student	388	0.75	0.09	0.54	1.05	0.57	0.004	0.39	0.84	0.70	0.07	0.48	1.03
Student engaged	402	0.72	0.04	0.52	0.99	0.72	0.06	0.51	1.00	0.72	0.08	0.50	1.05
Time reading	439	0.85	0.22	0.64	1.11	0.91	0.52	0.68	1.21	0.89	0.46	0.65	1.21
*Student report ( n* = 568)													
Did activities	567	0.76	0.16	0.51	1.11	0.69	0.07	0.46	1.04	0.61	0.02	0.40	0.93
Parent read handbook	567	0.75	0.21	0.48	1.17	0.11	0.65	0.70	1.77	0.86	0.54	0.54	1.38

Controlling for cohort, minority, first generation, and sex

**Table 6:** Logistic regression: odds ratios of associations between implementation and T2 (fall semester) alcohol/cannabis outcomes

	Binge drinking					Heavy episodic drinking			
*Predictor*	*n*	OR	*p*	Lower limit	Upper limit	OR	*p*	Lower limit	Upper limit
*Parent Report* (*n* = 437)									
Useful to student	388	0.73	0.06	0.54	1.01	0.82	0.33	0.58	1.20
Student engaged	402	0.62	0.002	0.46	0.84	0.73	0.09	0.51	1.05
Time reading	439	0.91	0.45	0.70	1.17	0.79	0.14	0.57	1.09
*Student report ( n* = 568)									
Did activities	567	0.64	0.02	0.44	0.92	0.78	0.26	0.51	1.20
Parent read handbook	567	0.77	0.20	0.51	1.15	0.57	0.02	0.38	0.92

Controlling for cohort, minority, first generation, and sex

In contrast, student report of engaging in handbook activities with a parent was less strongly and less consistently associated with lower odds of reporting use for alcohol (OR = 0.76, *p* = 0.16), cannabis (OR = 0.69, *p* = 07), simultaneous use (OR = 0.64, *p* = 0.02), binge drinking (OR = 0.78, *p* = 0.26), and heavy episodic drinking (OR = 0.82, *p* = 0.33). There was very little association of simply remembering that a parent read the handbook with any of the outcomes except heavy episodic drinking (OR = 0.57, *p* = 0.02).

#### Parent Contact, Parent Engagement, and Student Outcomes

In regression analyses, research assistant contact with parents predicted use of the handbook (*B* = 0.72, SE = 0.19*, p* < 0.001). In turn, parent engagement strongly predicted all substance use variables except heavy episodic drinking (see Supplementary materials). There was no direct effect of parent contact with outcomes, confirming our expectation that parent engagement was the proximal implementation step affecting student outcomes.

## Discussion

We reported results from the efficacy test of an intervention for parents of young adult students transitioning to college. Although some universities provide parents of matriculating students with information about substance use, there is little evidence-based guidance for parents on the importance of their role at this developmental stage. Our approach was to create a handbook that incorporated theory and research on the protective effects of clear parent communication, autonomy support, and involvement. Rather than using a didactic approach focused on substance misuse prevention, we provided parents with conversation starters and interactive exercises designed to foster meaningful conversations about the young adults’ values and their transition to independence. We hypothesized that by using the handbook, parents would meet the changing developmental needs of their students as they moved away from home, and that this in turn would decrease likelihood of students’ use of alcohol and cannabis in the high-risk period immediately following transition to college.

Data from the study support the hypothesis that use of the handbook had protective effects on student substance use behaviors. Effect sizes across different types of substance use were consistently related with outcomes and of similar magnitude, showing that students in the handbook condition were less likely to initiate use of alcohol or cannabis their first semester in college (odds ratios ranging from 0.45 to 0.56). Reported prevalence of past-30-day use increased substantially across all students who had reported substance use in spring of their senior year of high school, but prevalence was also consistently lower among students in the intervention than among control students during fall semester at college (odds ratios ranging from 0.67 to 0.74).

Multiple tests increase the likelihood of family-wise error, and two of the prevalence odds ratios had 1 in their confidence intervals. Thus we must be cautious about overinterpretation. However, the raw prevalence ranks are consistently lower in the intervention condition, as are the odds ratios, and there is a consistency of all odds ratios showing lower use in the intervention condition in the regression analyses. This pattern provides evidence that exposure to the intervention resulted in lower substance use among students whose parents used the handbook.

It is important to address the ways in which this test of our intervention expands on the pioneering work of Turrisi and his colleagues in the area of parent-based interventions intended to reduce college student substance use. First, the most widely cited evaluations of Turrisi’s intervention (Turrisi & Ray, 2010; Turrisi et al., 2001) did not include either random assignment to condition or baseline assessments of students’ behaviors. It is therefore difficult to directly compare results from the two evaluations. That being said, the most important commonality across both interventions is that each shows evidence of reducing heavy episodic drinking, a critical factor in reducing negative consequences from alcohol use. However, the current study highlights several important advances beyond those reported by Turrisi and colleagues. First, our results indicate that the intervention reduces students’ *initiation* of drinking, which is crucial given that inexperience may contribute to alcohol misuse and problematic consequences across the transition to college. Second*, *the* First Years Away from Home: Letting Go and Staying Connected* intervention is related to less cannabis use and a lower likelihood of initiating cannabis use. Finally, the handbook focuses on strengthening parenting skills rather than specifically targeting risk behaviors.

Implementation analyses showed that most parents read and used the handbook, that calls from trained research assistants increased the likelihood that they would do so, and that student substance use was associated with dosage in parent report of active *use* of the handbook (but not with just reading it) and their perception of student engagement and perceived usefulness. We interpret the stronger association of active engagement and perceived usefulness, as opposed to simple reading, as providing discriminant validation between passive vs. active use.

Our sample was limited to traditional college-aged first-year students at a residential public university; results may not generalize to nontraditional students or other settings. Our data included only binary gender designation; it is unknown if those identifying as non-binary or transgender would receive similar benefits from the intervention. Some parents may not have been fluent in English, and more broadly results may not generalize across cultures and ethnicities. The results of the present study are also limited to the first semester of college and may not generalize to future semesters. Strengths of the study include its theoretical foundation, randomization to condition, high implementation rates and sample retention, and the association of active vs. passive handbook use with intervention effects.

Our next steps are to test longer-term efficacy by examining intervention effects on trajectories of use and to explore mediating effects of parent-student relationship and communication characteristics and of implementation factors. We hope to conduct replication trials to test generalizability. We will also examine whether the booster texts throughout the academic year in the enhanced condition added value to the intervention and if effectiveness of the texts is associated with parenting practices. The handbook is potentially an effective way to engage parents, but sub-groups of parents (e.g., parents of minoritized students and of first-generation students) reported lower rates of engagement (Cooper et al., 2021). Research on sub-group acceptability, accessibility, and uptake is needed to help inform additions and adaptations to handbook content; currently, we are conducting such research for a Spanish-language adaptation of the handbook into video and printed format. Finally, we do not know to what extent the encouragement by the research assistants was a necessary element of that engagement. This, too, remains a question for future research.

From this study, we may conclude that parents remain influential in the lives of their students after they leave home for college and can help both to prevent students’ early initiation of substance use altogether and to minimize frequency of use, simultaneous use, and extreme use. This is important because students report numerous negative consequences from use of cannabis and alcohol (Caldeira et al., 2008; White & Hingson, 2013), especially from heavy episodic drinking (Fairlie et al., 2019), and recent research shows concerning consequences of co-use (Lee et al., 2020; Yurasek et al., 2017). Reducing increases in substance misuse and less heavy drinking may reduce negative consequences of substance misuse at the critical transition from high school to college (Sher & Rutledge, 2007; Small et al., 2011).

Results of the study are also important because parents continue to receive the message that they should back off and try not to be “helicopter parents.” A recent editorial in the New York Times by a mother who had just dropped her daughter off at college said “The campus psychologist had sent out a note to all parents of incoming freshmen, imploring us to limit contact and emphasizing that this includes texts. Apparently, this is a time for our children to ‘individuate and separate’” (Corrigan, 2021). Our study suggests instead that parents should indeed provide support for students’ growth and autonomy, but they should also continue to communicate regularly with their children and provide clear expectations and continued involvement and emotional support.

Because there is little opportunity for university administrators to interact with parents, they have been underutilized as partners in prevention. The theory-based, interactive handbook *First Years Away from Home: Letting Go and Staying Connected* provides an efficacious, low-cost method to provide evidence-based guidance to parents of college students.

## Supplementary Information

Below is the link to the electronic supplementary material.

Supplementary file1 (DOCX 177 kb)

Supplementary file2 (XLSX 19 kb)

Supplementary file2 (PDF 112 kb)

## Data Availability

The datasets analysed in the current study are available from the corresponding author on reasonable request.
