# Supplementary material for: “Letting Go and Staying Connected”: Substance Use Outcomes from a Developmentally Targeted Intervention for Parents of College Students
Source: Prev Sci. 2023 Mar 18;24(6):1174–86. doi: 10.1007/s11121-023-01520-6 (PMC10423701; doi:10.1007/s11121-023-01520-6)

**Supplementary Materials 1**

**Association of Parent Contact with Parent Engagement**

Outcome: Parent Use of handbook


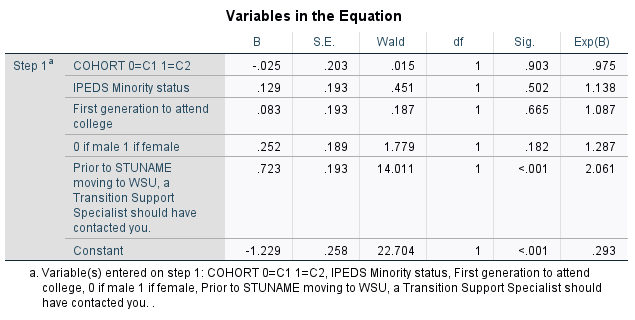


**2. Association of Parent Engagement with Student Outcomes**

ALCOHOL


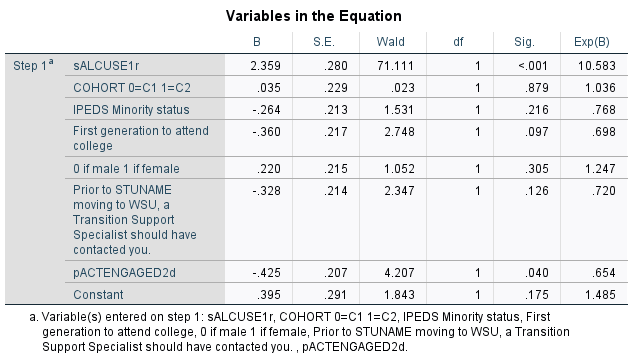


CANNABIS


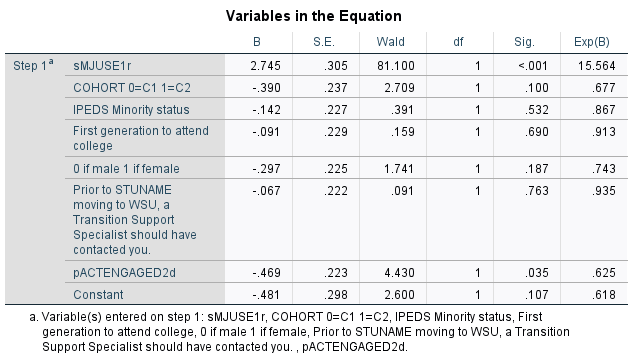


SIMULTANEOUS USE ALCOHOL and CANNABIS


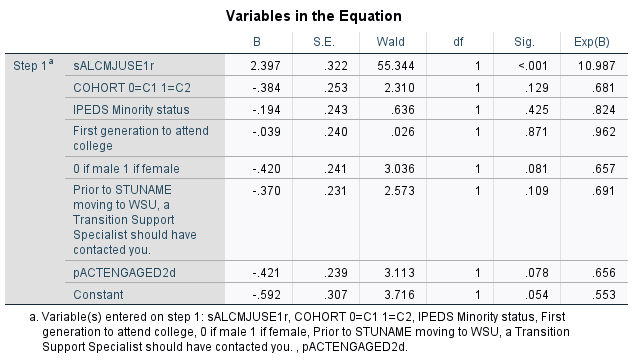


2 WK BINGE


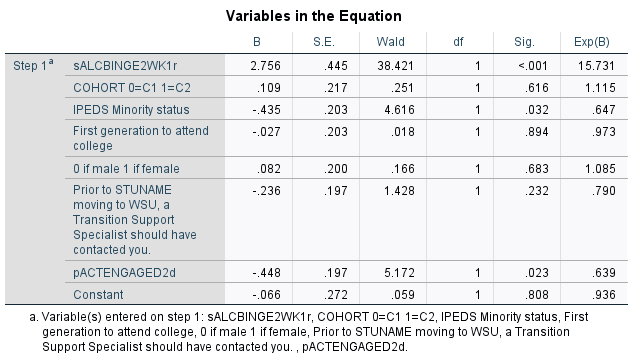


30 DAY HEAVY


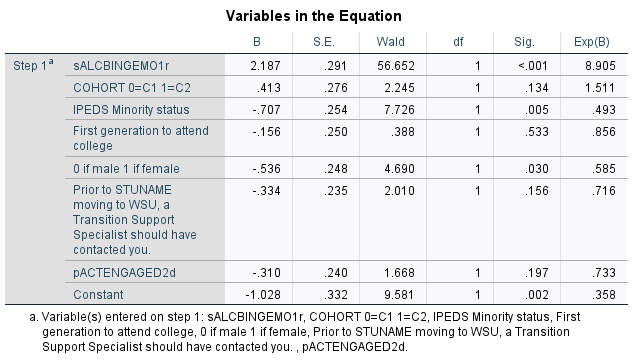

Supplement: Supplementary file 1 — Supplementary file1 (DOCX 177 kb) [file 11121_2023_1520_MOESM1_ESM.docx]
