# Supplementary figures and images for: “Letting Go and Staying Connected”: Substance Use Outcomes from a Developmentally Targeted Intervention for Parents of College Students
Source: Prev Sci. 2023 Mar 18;24(6):1174–86. doi: 10.1007/s11121-023-01520-6 (PMC10423701; doi:10.1007/s11121-023-01520-6)

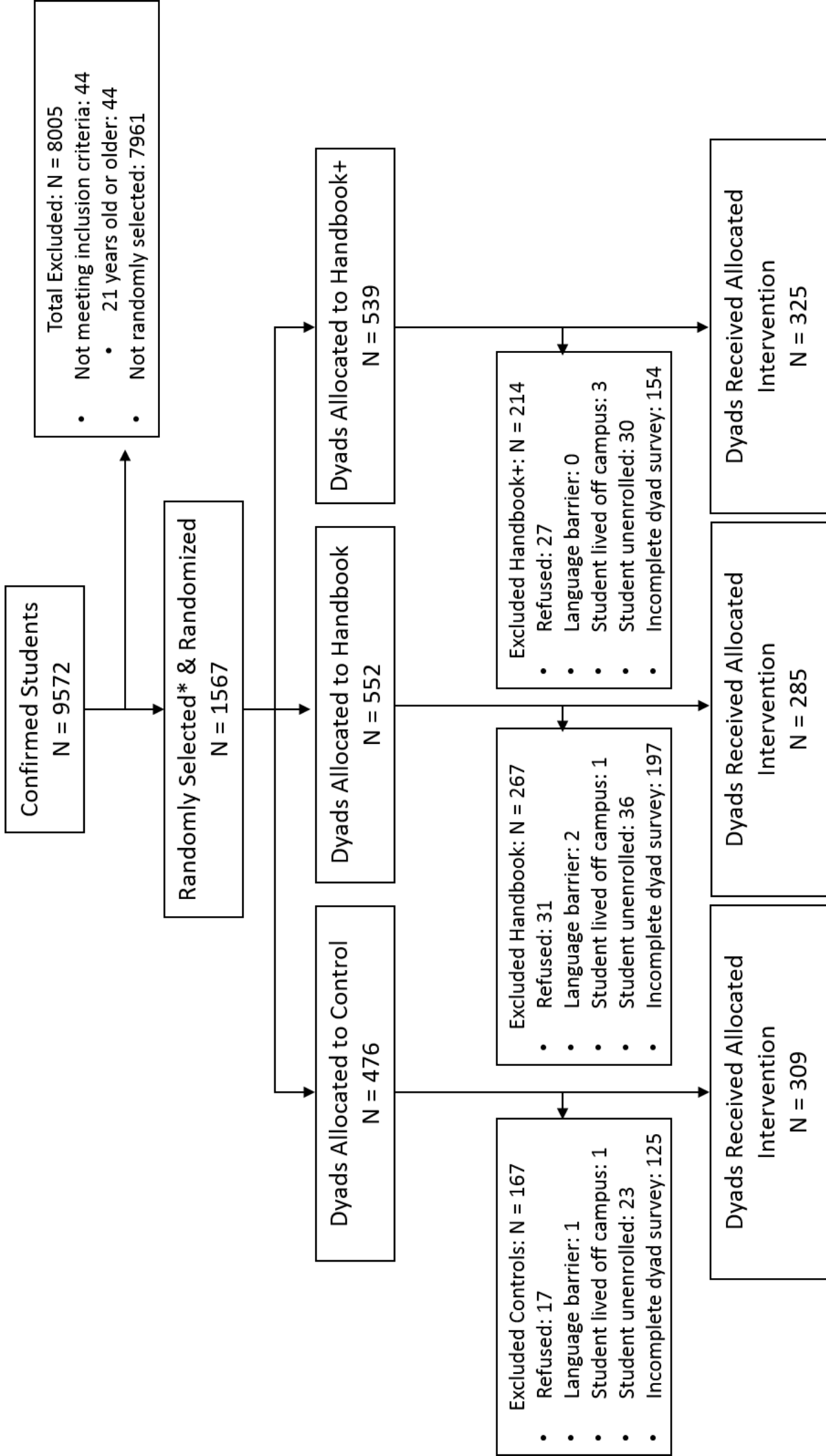

Supplement: Supplementary file 3 — Supplementary file2 (PDF 112 kb) [file 11121_2023_1520_MOESM3_ESM.pdf]
